# *Announcement*: Monitoring Selected National HIV Prevention and Care Objectives

**DOI:** 10.15585/mmwr.mm6629a6

**Published:** 2017-07-28

CDC monitors progress on selected national human immunodeficiency virus (HIV) prevention and care objectives using surveillance data (*1*) and has released two HIV care continuums for 2014: a diagnosis-based continuum and a prevalence-based continuum (*2*,*3*).

A diagnosis-based HIV continuum monitors key steps needed for a person living with diagnosed HIV infection to reach viral suppression, which leads to improved health outcomes and reduced risk for transmission to others. To determine a diagnosis-based HIV continuum, CDC uses the number of persons living with diagnosed HIV infection as the denominator. CDC monitors engagement in medical care and viral suppression in 38 jurisdictions that have complete reporting of CD4 and viral load laboratory results. Among persons living with diagnosed HIV infection at year-end 2014 in 38 jurisdictions, 73% received HIV medical care in 2014, 57% were retained in continuous care, and 58% were virally suppressed (*1*).

Because the first step in entering HIV care is receiving a diagnosis, CDC has also estimated an HIV prevalence-based continuum, which uses the estimated number of all persons living with diagnosed or undiagnosed HIV infection as the denominator. Among the estimated 1.1 million persons living with HIV infection in the United States in 2014, 85% had received a diagnosis (*1*). Extrapolating from 38 jurisdictions with complete reporting, an estimated 62% of persons living with HIV infection received HIV medical care in 2014, 48% were retained in continuous care, and 49% were virally suppressed (*2*).

More information is available in the Division of HIV/AIDS Prevention report and accompanying fact sheet and slide set (*1*–*3*).

## References

[1] National Center for HIV/AIDS. Viral Hepatitis, STD, and TB Prevention. Monitoring selected national HIV prevention and care objectives by using HIV surveillance data—United States and 6 dependent areas, 2015. HIV surveillance supplemental report, vol. 22, no.2. Atlanta, GA: US Department of Health and Human Services, CDC, National Center for HIV/AIDS, Viral Hepatitis, STD, and TB Prevention; 2017. https://www.cdc.gov/hiv/pdf/library/reports/surveillance/cdc-hiv-surveillance-supplemental-report-vol-22-2.pdf

[2] National Center for HIV/AIDS. Viral Hepatitis, STD, and TB Prevention. Selected national HIV prevention and care outcomes. Atlanta, GA: US Department of Health and Human Services, CDC, National Center for HIV/AIDS, Viral Hepatitis, STD, and TB Prevention; 2017. https://www.cdc.gov/hiv/library/slideSets/index.html

[3] National Center for HIV/AIDS. Viral Hepatitis, STD, and TB Prevention. Selected national HIV prevention and care outcomes in the United States. Atlanta, GA: US Department of Health and Human Services, CDC, National Center for HIV/AIDS, Viral Hepatitis, STD, and TB Prevention; 2017. https://www.cdc.gov/hiv/pdf/library/factsheets/cdc-hiv-national-hiv-care-outcomes.pdf

